# Presence of Chronic Obstructive Pulmonary Disease (COPD) Impair Survival in Lung Cancer Patients Receiving Epidermal Growth Factor Receptor-Tyrosine Kinase Inhibitor (EGFR-TKI): A Nationwide, Population-Based Cohort Study

**DOI:** 10.3390/jcm8071024

**Published:** 2019-07-12

**Authors:** Chia-Che Wu, Kun-Ming Rau, Wei-Chieh Lee, Meng-Che Hsieh, Jia-Sin Liu, Yen-Yang Chen, Harvey Yu-Li Su

**Affiliations:** 1 Division of Hematology Oncology, Department of Internal Medicine, Kaohsiung Chang Gung Memorial Hospital and Chang Gung University, College of Medicine, Kaohsiung 83301, Taiwan; 2 Department of Hematology Oncology, E-Da Cancer Hospital and I-Shou University, College of Medicine, Kaohsiung 82445, Taiwan; 3 Division of Cardiology, Department of Internal Medicine, Kaohsiung Chang Gung Memorial Hospital and Chang Gung University, College of Medicine, Kaohsiung 83301, Taiwan; 4 Department of Public Health, College of Health Science, Kaohsiung Medical University, Kaohsiung, Kaohsiung 80708, Taiwan; 5 Clinical Trial Center, Kaohsiung Chang Gung Memorial Hospital, Kaohsiung 83301, Taiwan

**Keywords:** COPD, EGFR-TKI, lung cancer, survival, Taiwan

## Abstract

The emergence of epidermal growth factor receptor-tyrosine kinase inhibitor (EGFR-TKI) caused a paradigm shift in the treatment of non-small cell lung cancer (NSCLC). Although several clinicopathologic factors to predict the response to and survival on EGFR-TKI were recognized, its efficacy has not been confirmed for patients with underlying pulmonary disease, such as chronic obstructive pulmonary disease (COPD). We conducted the study to evaluate the impact of COPD on survival for NSCLC patients that underwent EGFR-TKI treatment. The nationwide study obtained clinicopathologic data from the National Health Insurance Research Database in Taiwan between 1995 and 2013. Patients receiving EGRR-TKI were divided into COPD and non-COPD groups, and adjusted for age, sex, comorbidities, premium level and cancer treatments. Overall survival (OS) and progression-free survival (PFS) were calculated by Kaplan–Meier analysis. In total, 21,026 NSCLC patients were enrolled, of which 47.6% had COPD. After propensity score (PS) matching, all covariates were adjusted and balanced except for age (*p* < 0.001). In the survival analysis, the median OS (2.04 vs. 2.28 years, *p* < 0.001) and PFS (0.62 vs. 0.69 years, *p* < 0.001) of lung cancer with COPD were significantly worse than those without COPD. Lung cancer patients on EGFR-TKI treatment had a worse survival outcome if patients had pre-existing COPD.

## 1. Introduction

Lung cancer is a global common malignancy, and accounts for the leading cause of cancer-related death in the USA, Asia and Taiwan [1,2]. Although much progress in treatment of metastatic non-small cell lung cancer (NSCLC) has been made in recent decades, the 5-year survival of NSCLC in Taiwan remains poor (all stages, 16.3%). With increasing knowledge about oncogenic mutation of NSCLC, several effective therapeutic opinions have been developed, including a specific antibody to the epidermal growth factor receptor (EGFR) and anaplastic lymphoma kinase (ALK). For NSCLC harboring EGFR driver mutation, the current standard of treatment in the first-line setting is EGFR tyrosine kinase inhibitor (TKI), either first-generation (gefitinib or erlotinib) or second-generation TKI (afatinib) [3,4,5,6]. A recent study, analyzing six randomized clinical trials, compared EGFR-TKI with conventional chemotherapy in the first-line setting and showed a significant increment in progressive-free survival (PFS) from 5.6 months with chemotherapy to 11.0 months with EGFR-TKI [7]. Although the presence of activating EGFR mutation (del19 or L858R) precisely predicts good response to EGFR-TKI, there are, however, several clinical factors showing various responses to EGFR-TKI, and the underlying mechanism still remains unknown [8]. 

Lung cancer patients are generally older, and beyond all doubt, associated with high prevalence of coexisting comorbidity such as chronic obstructive pulmonary disease (COPD), congestive heart failure, diabetes mellitus and cardiovascular disease [9]. COPD is considered as a cigarette-related chronic pulmonary inflammatory disease, which shares a common pathogenic pathway with lung cancer. Several studies showed COPD increased the risk for NSCLC, notably independent to patient age or extent of cigarette exposure [10,11,12]. Moreover, the relationship between COPD and overall survival of NSCLC has drawn much research interests, although the result still remains inconclusive. A recent meta-analysis, consisting of 26 qualified observational studies, showed that presence of COPD highly correlated with poor survival in localized and resected NSCLC [13]. However, among patients that received chemotherapy for metastatic NSCLC, several studies showed inconclusive results in discussing the survival impact of COPD in metastatic NSCLC [14,15]. Also, there is not enough evidence to explore the role of COPD in patients treated with EGFR-TKI. Therefore, we conducted a large-scale, nationwide, population-based study by using the National Health Insurance Research Database (NHIRD) to investigate the prognostic role of COPD in metastatic NSCLC patients receiving EGFR-TKI. 

## 2. Material and Methods

### 2.1. Data Source and Study Population

In the population-based cohort study, we used the Taiwan National Health Insurance Research Database (NHIRD) as the data source. The National Health Insurance (NHI) program was initiated in 1995, providing universal and compulsory health care that covers >99% of all residents (23.7 million people) in Taiwan. The NHIRD consists of unrecognizable longitudinal health care claims data, including sex, date of birth, medical institutions, medical or surgical procedures, and all information regarding inpatient and outpatient visits. The diagnoses were coded in accordance with the International Classification of Diseases, Ninth Revision, Clinical Modification (ICD-9-CM). The Bureau of NHI requires registration of patients with “catastrophic illness”, such as end-stage renal disease, congenital abnormalities, autoimmune diseases and all types of cancers. The vulnerable beneficiaries who were issued with a Catastrophic Illness Certificate (CIC) will be exempted from copayments for the corresponding medical services. Approval of CIC requires strict evaluation by experts in the Bureau of the NHI. The NHIRD encrypts all recognizable information of beneficiaries, and thus provides investigators anonymous data to analyze, with strict confidential guidelines. 

### 2.2. Study Cohort

We identified all lung cancer patients (ICD-9-CM: 162) using either hospitalization or outpatient diagnoses between 1995 and 2013. The date of lung cancer diagnosis, either by clinical imaging or pathologic confirmation, was set as the index date. To explore our hypothesis, we confined study cohort to lung cancer patients that underwent EGFR-TKI (gefitinib or erlotinib) and divided it into two subgroups based on the presence of COPD (ICD-9-CM: 491, 492, 496). We further performed propensity score matching to balance the intergroup difference of covariates. 

In Taiwan, the application and use of EGFR-TKI for third-line therapy of chemotherapy refractory NSCLC by NHI reimbursement has been approved since November 2004 (gefitinib). In November 2013, gefitinib and erlotinib got approved and reimbursed as first-line therapy for lung adenocarcinoma harboring active EGFR mutations. Physicians must evaluate the treatment response of EGFR-TKI every three months by imaging tools. Once progressive disease was documented by imaging, re-application of EGFR-TKI was not allowed and be turned down by the NHI.

### 2.3. Demographic Covariates and Comorbidities

The demographic characteristics of age, sex, income for insurance payment and comorbidities using the Charlson comorbidity index (CCI), which included congestive heart failure (CHF), diabetes mellitus (DM), pneumonia and sepsis, were identified and considered as covariates. Patients were classified in three levels of monthly income: NTD < 15,840, NTD 15,841–25,000, and NTD > 25,000. Anti-cancer treatments, including all kinds of chemotherapy (CT) regimens, radiotherapy (RT) and EGFR-TKI, were also included the study. EGFR-TKI and chemotherapy can be used alternatively, and we also showed the treatment sequence prior to EGFR-TKI. Concomitant drugs, including statins, non-steroidal anti-inflammatory drugs (NSAID), aspirin, anti-hypertension and steroids, were also listed and compared between the two cohorts. 

### 2.4. Propensity Score Matching

We performed propensity score matching to eliminate intergroup selection bias. The propensity score (PS) is defined as the conditional probability of lung cancer patients with or without COPD given in some measurable covariates. A non-parsimonious multivariable logistic regression model was used for calculating PS. The covariates in the 1:1 PS matching model were adjusted for age, gender, monthly income, various comorbidities and many types of chemotherapy, radiotherapy and EGFR-TKI treatment. 

### 2.5. Endpoints

The main endpoint of the study was overall survival (OS). OS was defined as the time period from the index date to any cause of death or censored at the last follow-up visit. The second endpoint was progression-free survival (PFS), which was defined as the period between administration of EGFR-TKI and the end of dosing. Physicians may discontinue EGFR-TKI treatment based on imaging (CT, MRI or chest X-ray) progression, abnormal physical examination (e.g., enlarged lymph node), remarkedly increased tumor markers or toxicity. Although our study was not designed as a prospective study with a scheduled imaging survey, the PFS could be roughly reliable because of strict scrutiny of EGFR-TKI application. 

### 2.6. Statistical Analysis

The intergroup differences in demographic variables, comorbidities and treatments were compared by *t* tests for continuous variables and by chi-squared tests of Fisher’s exact test for categorical variables. We performed survival analysis for OS and PFS by Kaplan–Meier analysis and compared significance by the log-rank test. To calculate the hazard ratio (HR) of the risk of death and progression-free survival, we used Cox proportional hazard regression analysis. An adjusted model was conducted by adjusting age, gender, premium level, comorbidities, anti-cancer treatments and EGFR-TKIs in a multivariate analysis. All analyses were conducted using SAS statistical software analysis (Version 9.4; SAS institute, Cary, NC, USA) 

## 3. Results

### 3.1. Patient Characteristics

**Figure 1:**
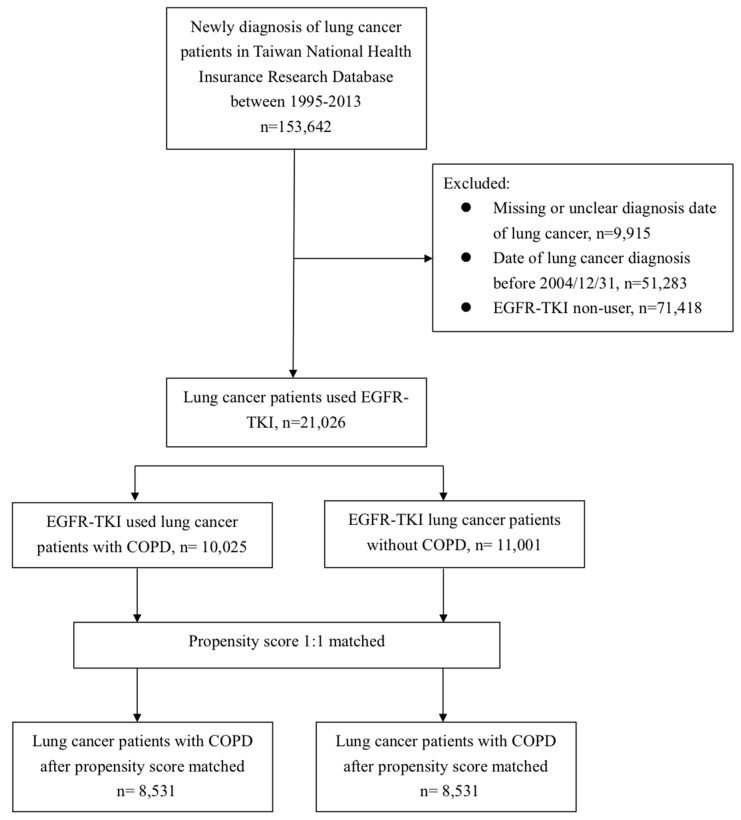
Flow diagram of patient’s enrollment.

In total, we screened 153,642 lung cancer patients initially. To identify the EGFR-TKI users, we excluded 9915 individuals due to missing or unclear diagnosis dates, 51,283 individuals due to diagnosis dates before EGFR-TKI reimbursement, and 71,417 patients without any records of EGFR-TKI administration. Figure 1 demonstrate the CONSORT diagram and patients flow in detail. At last, we identified a total of 21,026 lung cancer patients treated with EGFR-TKI during the study period. The detailed demographic information of whole EGFR-TKI users is shown in Table 1. Among them, 47.6% of patients had the diagnosis of COPD, 4% of congestive heart failure, 19% of diabetes mellitus, 16% of pneumonia and 0.9% of them had the diagnosis of sepsis. For patients receiving EGFR-TKI, 47% of all were erlotinib, 62% of them were gefitinib and 9% of all received both erlotinib and gefitinib. Approximately two thirds of patients (68%) underwent chemotherapy, and the mostly commonly used regimens were gemcitabine (41%), vinorelbine (38%), docetaxel (34%), pemetrexed (33%) and cisplatin (20%). 

In the model before PS matching, there were statistically significant differences between COPD and non-COPD groups, including age (*p* < 0.001), sex (*p* < 0.001), premium level (*p* < 0.001), comorbidities (*p* < 0.001), anti-neoplastic agents (*p* < 0.001) and concomitant drugs (*p* < 0.001). After 1:1 PS adjustment, there were 8531 individuals allocated equally in both COPD and non-COPD groups. Although the mean age of COPD patients was slightly older than non-COPD patients (65.6 vs. 64.9 years, *p* < 0.001), there were no other significant intergroup differences regarding to gender, premium level, comorbidity and use of anti-neoplastic agents (Table 1). 

### 3.2. PFS and OS in COPD and non-COPD EGFR-TKI Users

**Figure 2:**
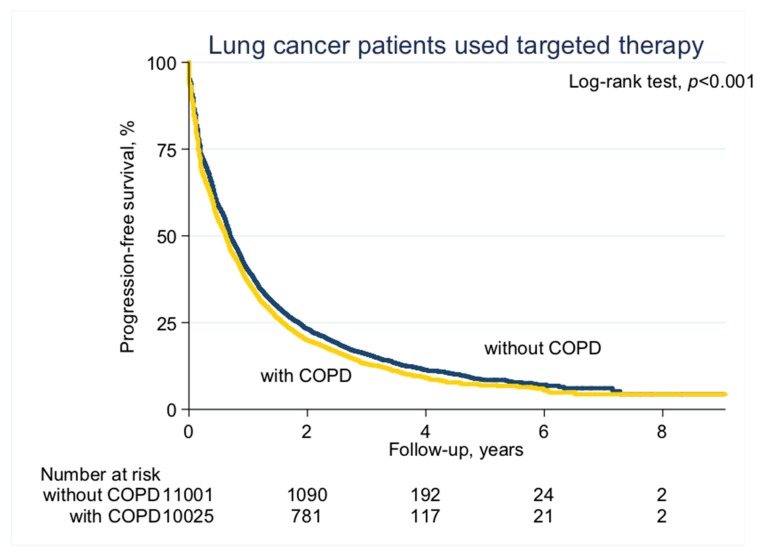
Progression-free survival curve of chronic obstructive pulmonary disease (COPD) and non-COPD cohorts (before propensity score (PS) matching).

**Figure 3:**
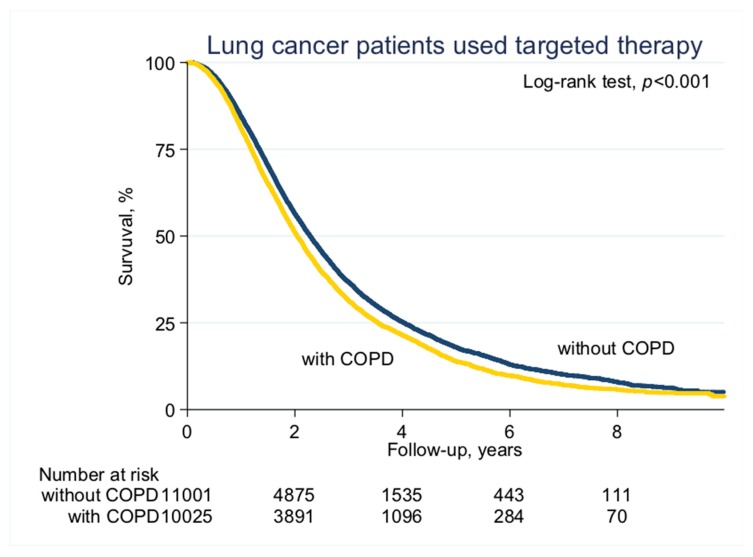
Overall survival curve of COPD and non-COPD cohorts (before PS matching).

**Figure 4:**
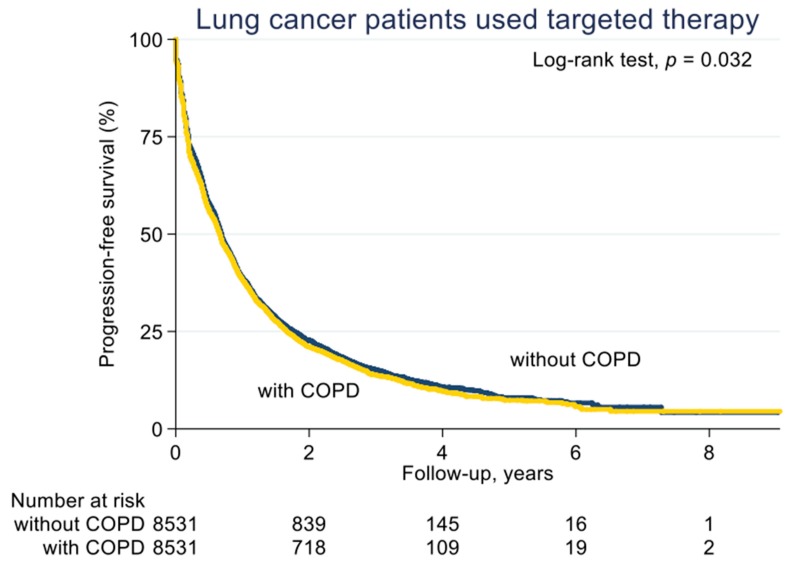
Progression-free survival curve of COPD and non-COPD cohorts (after PS matching).

**Figure 5:**
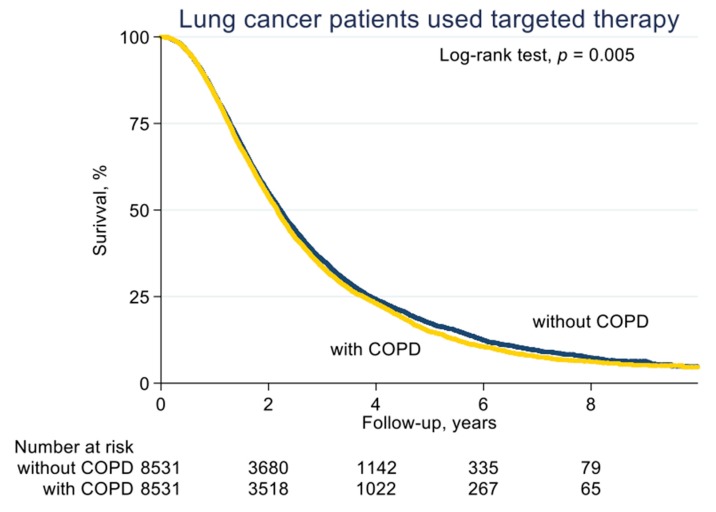
Overall survival curve of COPD and non-COPD cohorts (after PS matching).

With a median follow-up of 2.1 years (standard deviation (SD), 1.6 years), the median PFS on EGFR-TKI calculated by Kaplan–Meier analysis in the COPD cohort (before PS matching) was 0.62 years, significantly shorter than the non-COPD cohort (0.69 years, *p* < 0.001; Figure 2). Of note, non-COPD lung cancer patients had a significantly longer median OS than the COPD cohort (2.28 vs. 2.04 years, *p* < 0.001; Figure 3). We also estimated the PFS and OS difference in the model after PS matching. Unsurprisingly, the COPD cohort had a significantly worse PFS (0.43 vs. 0.47 years; *p* = 0.032; Figure 4) and OS (1.71 vs. 1.75 years, *p* = 0.005; Figure 5) compared to the non-COPD cohort.

### 3.3. Comparison of HRs of Death for All Covariates

Table 2 shows the risk of death in COPD and non-COPD cohorts comparing all clinical covariates. Being male (HR 1.33, 95% CI 1.29–1.38, *p* < 0.001), a low premium level of <15,840 NTD (HR 1.22, 95% CI 1.16–1.30, *p* < 0.001), CHF (HR 1.16, 95% CI 1.08–1.26, *p* < 0.001), DM (HR 1.12, 95% CI 1.07–1.17, *p* < 0.001), pneumonia (HR 1.25, 95% CI 1.19–1.30, *p* < 0.001), sepsis (HR 1.44, 95% CI 1.22–1.70, *p* < 0.001) and NSAID (HR 1.18, 95% CI 1.10–1.27, *p* < 0.001) were the significant risks of death in the crude univariate analysis. Patients that underwent EGFR-TKI, radiotherapy (RT) or chemotherapy (CT), irrespective of any regimen, had significantly reduced risks of death. Concomitant medications such as statin (HR 0.86, 95% CI 0.83–0.90, *p* < 0.001), aspirin (HR 0.94, 95% CI 0.91–0.98, *p* < 0.001) and anti-hypertension agents (HR 1.33, 95% CI 1.29–1.38, *p* < 0.001) were significantly associated with a reduction in mortality. 

In the adjusted model which was modified for age, sex, premium level, comorbidities, anti-neoplastic agents, cancer treatments, CT regimen before EGFR-TKI and concomitant drugs, lung cancer patients with COPD still remained at a higher risk of death (HR 1.05, 95% CI 1.01–1.08, *p* = 0.012). Administration of EGFR-TKI in the adjusted model showed decreased all cause of mortality in a greater magnitude than in the crude model (HR 0.56 for erlotinib, 95% CI 0.53–0.60, *p* < 0.001). Other independent prognostic factors in the adjusted model were similar to those in crude model. 

Lastly, after PS matching and adjustment for all competitive confounding factors, COPD (HR 1.04, 95% CI 1.00–1.08, *p* = 0.03), male (HR 1.32, 95% CI 1.27–1.38, *p* < 0.001), low premium level (HR 1.18, 95% CI 1.13–1.23, *p* < 0.001), CHF (HR 1.13, 95% CI 1.03–1.25, *p* = 0.01), DM (HR 1.14, 95% CI 1.09–1.20, *p* < 0.001), pneumonia (HR 1.19, 95% CI 1.13–1.25, *p* < 0.001), sepsis (HR 1.24, 95% CI 1.03–1.49, *p* = 0.02), >1 CT regimen before EGFR-TKI (HR 1.13, 95% CI 1.06–1.20, *p* < 0.001), NSAID (HR 1.15, 95% CI 1.06–1.25, *p* = 0.001) and steroid (HR 1.07, 95% CI 1.02–1.12, *p* = 0.01) were significantly associated with an increased risk of death. A reduced risk of death was observed in erlotinib (HR 0.56, 95% CI 0.52–0.60, *p* < 0.001), gefitinib (HR 0.55, 95% CI 0.51–0.59, *p* < 0.001), pemetrexed (HR 0.79, 95% CI 0.75–0.82, *p* < 0.001), vinorelbine (HR 0.83, 95% CI 0.80–0.86, *p* < 0.001), cisplatin (HR 0.92, 95% CI 0.87–0.97, *p* = 0.001), paclitaxel (HR 0.89, 95% CI 0.84–0.94, *p* < 0.001), carboplatin (HR 0.88, 95% CI 0.81–0.95, *p* = 0.001), statin (HR 0.85, 95% CI 0.80–0.89, *p* < 0.001), aspirin (HR 0.85, 95% CI 0.81–0.89, *p* < 0.001) and anti-hypertension agents (HR 0.84, 95% CI 0.81–0.88, *p* < 0.001). 

### 3.4. Comparison of HRs of Progression

Table 3 shows the HRs of progression-free survival in all covariates. In brief, COPD remained a significant impairment of PFS (HR 1.11, 95% CI 1.05–1.15, *p* < 0.001) in the unadjusted model and in the PS matching model (HR 1.05, 95% CI 1.02–1.09, *p* = 0.006).

## 4. Discussion

In this large scale, nationwide, population-based cohort study, we demonstrated that COPD is a crucial prognostic factor for lung cancer patients treated with EGFR-TKI. We believe that this is the first study to emphasize the clinical role of COPD in selecting therapeutic EGFR-TKI for NSCLC patients. 

Several anecdotal studies investigated the relationship between COPD and lung cancer. Both diseases share common causative risks and pathologic pathways [16]. The UK General Practice Research Database, which enrolled more than 4 million patients within a 14-year period, showed that the 3-year survival for lung cancer patients with COPD was almost half of the general population (15% vs. 26%; *p* < 0.01) [17]. Other studies reported that the survival of lung cancer with COPD were significantly worse in different situations [18,19]. A recent meta-analysis focused on the impact of COPD on post-resection survival of lung cancer patients, showing that patients with COPD had higher post-operation complications (pneumonia and prolonged mechanical ventilation) and poor overall survival [18]. The reason of inferior survival was likely attributed to inevitable deterioration of pulmonary function following lung resection, and COPD patients with known bad pulmonary function had poor tolerance to surgery, leading to an increased rate of post-operation pneumonia and cancer recurrence [20]. 

In the era of targeting therapy for lung cancer, EGFR-TKI have been proven as the pivotal therapeutic role for NSCLC harboring an active EGFR mutation in terms of response rate, PFS and OS [5,6,7]. Several studies emphasized that the frequency of EGFR mutation increased with several clinicopathologic factors, including adenocarcinoma, never smoker and being female [21]. As COPD is a well-known smoking-related disease, the incidence of an EGFR mutation in COPD patients with lung cancer might be lower than in non-COPD patients. Lim et al. reported that COPD was independently associated with a lower prevalence of EGFR mutation (odd ratio 0.197; 95% CI 0.065–0.600; *p* < 0.004), and the EGFR mutation rate conversely decreased with increased severity of COPD (*p* = 0.001) [22]. Importantly even in non-smoking patients, the EGFR mutation rate in patients with COPD was significantly lower than those without COPD (*p* = 0.001). Another study led by Takeda et al. showed 25% EGFR mutation in lung cancer without emphysema and 9% in those with emphysema [23]. As active EGFR mutation is the critical factor to predict the response and survival outcome for patients that underwent EGFR-TKI treatment, it is unsurprisingly that COPD patients might had poor treatment efficacy on EGFR-TKI because of inherited low EGFR mutation frequency [24]. 

Beyond the EFGR pathway, several alternative pathways between COPD and lung cancer were also discussed. Xiao et al. performed a comprehensive whole-genome and exome sequencing study to evaluate the different mutation in lung adenocarcinoma with and without COPD [25]. Not surprisingly that prevalence of EGFR mutation was higher in patients without COPD. Of note, a novel low density-lipoprotein receptor-related protein 1B (LRP1B) mutation was identified and significantly increased prevalence in lung cancer with COPD. The *LRP1B* gene is a putative tumor suppressor gene encoding an endocytic LDL-family receptor [26]. Some studies reported that deregulations of the *LRP1B* gene are associated with resistance to chemotherapy and worse survival in cancer [27,28]. Other studies discovered several mechanisms mediating lung cancer development in patients with COPD, such as telomere shortening [29,30], oxidative stress [31] and chronic inflammation [32]. Although increasing understandings of carcinogenesis and genomic pathways of COPD lung cancer have been made, there still no available targeting therapy directly to treat these devastated patients. 

Our study had some limitations. First, and the most important, the retrospective cohort study lacked evidence of EGFR mutation, which might cause an imbalanced distribution between COPD and non-COPD groups. The bias could be minimized by the high prevalence of EGFR mutation in Taiwan and the administrative process for first-line EGFR-TKI treatment by the NHI. The prevalence of EGFR mutation in Taiwan was much higher (40.8% to 62.1%) than that in Western countries, indicating that more than half of all lung cancers in Taiwan should be effectively treated with EGFR-TKI [33,34,35]. In addition, application for first-line EGFR-TKI treatment required attachment of EGFR mutation reports for administrative review by the NHI policy. In our study, 35% of all enrolled patients underwent first-line EGFR-TKI treatment in both cohorts, indicating that at least one third of patients had documented EGFR mutation and were allocated equally. Second, although the severity of COPD could be identified, it was not correlated with other comorbidities and survival. Despite this, we adjusted all possible confounding factors and performed PS matching, but the difference of age between the two cohorts still could not be eliminated and could therefore be a confounder. 

In conclusion, our study highlighted that lung cancer patients with COPD had a poorer survival outcome than those without COPD when treated with EGFR-TKI. Further research, either prospective randomized controlled trials or basic molecular studies, are needed to validate this finding. 

## Figures and Tables

**Table 1:** The demographic characteristics of lung cancer patients with epidermal growth factor receptor-tyrosine kinase inhibitor (EGFR-TKI).

	Before Propensity Score Matched			After Propensity Score Matched		
	With COPD	Without COPD	*p* Value	With COPD	Without COPD	*p* Value
*n*	10,025	11,001		8531	8531	
Age (mean, SD), years	67.6 (11.8)	62 (12.1)	<0.001	65.6 (11.3)	64.9 (11.4)	<0.001
Gender						
Male	5167 (51.5)	4804 (43.7)	<0.001	4091 (48.0)	4030 (47.2)	0.35
Female	4858 (48.5)	6197 (56.3)	<0.001	4440 (52.0)	4501 (52.8)	0.35
Premium level (NTD)						
>25,000	3588 (35.8)	4934 (44.9)	<0.001	3373 (39.5)	3480 (40.8)	0.09
25000–15840	4510 (45.0)	4313 (39.2)	<0.001	3657 (42.9)	3567 (41.8)	0.16
<15,840	1179 (11.8)	1027 (9.3)	<0.001	883 (10.4)	858 (10.1)	0.53
Low	748 (7.5)	727 (6.6)	0.016	618 (7.2)	626 (7.3)	0.81
Comorbidity						
CHF	662 (6.6)	343 (3.1)	<0.001	304 (3.6)	338 (4.0)	0.17
DM	2007 (20.0)	1835 (16.7)	<0.001	1677 (19.7)	1630 (19.1)	0.36
Pneumonia	2207 (22.0)	1571 (14.3)	<0.001	1382 (16.2)	1451 (17.0)	0.16
Sepsis	107 (1.1)	91 (0.8)	0.07	76 (0.9)	77 (0.9)	0.94
CCI score	4.7 (2.7)	4.2 (2.8)	<0.001	4.4 (2.6)	4.4 (2.9)	0.99
Anti-cancer agents						
Erlotinib	4770 (47.6)	4990 (45.4)	0.001	4004 (46.9)	3991 (46.8)	0.84
Gefitinib	6071 (60.6)	7083 (64.4)	<0.001	5278 (61.9)	5302 (62.1)	0.71
Erlotinib + Gefitinib	816 (8.1)	1072 (9.7)	<0.001	751 (8.8)	762 (8.9)	0.77
Gemcitabine	3962 (39.5)	4689 (42.6)	<0.001	3547 (41.6)	3546 (41.6)	0.99
Docetaxel	3321 (33.1)	3857 (35.1)	0.003	2965 (34.8)	2947 (34.5)	0.77
Pemetrexed	3070 (30.6)	3864 (35.1)	<0.001	2843 (33.3)	2845 (33.3)	0.99
Vinorelbine	3894 (38.8)	4113 (37.4)	0.03	3262 (38.2)	3219 (37.7)	0.50
Cisplatin	1852 (18.5)	2487 (22.6)	<0.001	1737 (20.4)	1764 (20.7)	0.61
Paclitaxel	1256 (12.5)	1498 (13.6)	0.019	1120 (13.1)	1136 (13.3)	0.72
Carboplatin	638 (6.4)	609 (5.5)	0.011	543 (6.4)	519 (6.1)	0.45
Cancer treatment						
CT + RT	3587 (35.8)	4626 (42.1)	<0.001	3335 (39.1)	3333 (39.1)	0.99
CT	6495 (64.8)	7664 (69.7)	<0.001	5802 (68.0)	5805 (68.0)	0.99
RT	4754 (47.4)	5914 (53.8)	<0.001	4302 (50.4)	4352 (51.0)	0.44
Without CT or RT	2363 (23.6)	2049 (18.6)	<0.001	1762 (20.7)	1707 (20.0)	0.30
CT regimens before EGFR-TKI						
0	3627 (36.2)	3956 (36.0)	0.74	3013 (35.3)	3043 (35.7)	0.63
1	2705 (27.0)	2880 (26.2)	0.19	2271 (26.6)	2249 (26.4)	0.70
≥2	3693 (36.8)	4165 (37.9)	0.13	3247 (38.1)	3239 (38.0)	0.90
Concomitant drug						
Statin	2151 (21.5)	1765 (16.0)	<0.001	1692 (19.8)	1629 (19.1)	0.22
NSAID	9480 (94.6)	9964 (90.6)	<0.001	8000 (93.8)	7956 (93.3)	0.17
Aspirin	3202 (31.9)	2332 (21.2)	<0.001	2304 (27.0)	2215 (26.0)	0.12
Anti-HTN	7265 (72.5)	6403 (58.2)	<0.001	5832 (68.4)	5690 (66.7)	0.02
Steroids	7820 (78.0)	8029 (73.0)	<0.001	6509 (76.3)	6462 (75.7)	0.40
Propensity score	0.6236 (0.4845)	0.5379 (0.4986)	<0.001	0.4859 (0.1201)	0.4778 (0.1266)	<0.001

Abbreviations: EGFR, epidermal growth factor receptor; TKI, tyrosine kinase inhibitor; SD, standard deviation; COPD, chronic obstructive pulmonary disease; NTD, new Taiwan dollar; CHF, congestive heart failure; DM, diabetes mellitus; CCI, Charlson comorbidity index; CT, chemotherapy; RT, radiotherapy; NSAID, non-steroidal anti-inflammatory drug; and HTN, hypertension.

**Table 2:** Comparison of the hazard ratios (HRs) of death for all clinical variables.

	Crude Model		Adjusted Model ^a^		Propensity Score Matched Adjusted Model	
	HR (95% CI)	*p* Value	HR (95% CI)	*p* Value	HR (95% CI)	*p* Value
All-cause mortality						
COPD	1.16 (1.12–1.20)	<0.001	1.05 (1.01–1.08)	0.012	1.04 (1.00–1.08)	0.033
Erlotinib	0.87 (0.84–0.90)	<0.001	0.56 (0.53–0.60)	<0.001	0.56 (0.52–0.60)	<0.001
Gefitinib	0.87 (0.84–0.90)	<0.001	0.55 (0.52–0.59)	<0.001	0.55 (0.51–0.59)	<0.001
Age (mean, SD), years	1.01 (1.01–1.01)	<0.001	1.01 (1.00–1.01)	<0.001	1.01 (1.00–1.01)	<0.001
Gender						
Male vs. Female	1.33 (1.29–1.38)	<0.001	1.33 (1.29–1.38)	<0.001	1.32 (1.27–1.38)	<0.001
Premium level (NTD)						
>25,000	1.0 (reference)		1.0 (reference)		1.0 (reference)	
25,000–15,840	1.16 (1.09–1.24)	<0.001	1.21 (1.12–1.30)	<0.001	1.17 (1.09–1.27)	<0.001
<15,840	1.22 (1.16–1.30)	<0.001	1.15 (1.08–1.22)	<0.001	1.13 (1.05–1.21)	<0.001
Low	1.20 (1.16–1.24)	<0.001	1.20 (1.15–1.24)	<0.001	1.18 (1.13–1.23)	<0.001
Comorbidity						
CHF	1.16 (1.08–1.26)	<0.001	1.10 (1.02–1.19)	0.016	1.13 (1.03–1.25)	0.013
DM	1.12 (1.07–1.17)	<0.001	1.15 (1.10–1.21)	<0.001	1.14 (1.09–1.20)	<0.001
Pneumonia	1.25 (1.19–1.30)	<0.001	1.19 (1.14–1.24)	<0.001	1.19 (1.13–1.25)	<0.001
Sepsis	1.44 (1.22–1.70)	<0.001	1.17 (0.99–1.38)	0.06	1.24 (1.03–1.49)	0.022
CCI score	1.05 (1.04–1.05)	<0.001	1.05 (1.04–1.05)	<0.001	1.05 (1.04–1.05)	<0.001
Anti-neoplastic agents						
Gemcitabine	0.94 (0.91–0.97)	<0.001	1.01 (0.97–1.05)	0.60	1.00 (0.95–1.04)	0.99
Docetaxel	0.89 (0.86–0.92)	<0.001	0.98 (0.94–1.02)	0.26	0.98 (0.93–1.02)	0.31
Pemetrexed	0.69 (0.67–0.72)	<0.001	0.79 (0.76–0.82)	<0.001	0.79 (0.75–0.82)	<0.001
Vinorelbine	0.84 (0.81–0.87)	<0.001	0.85 (0.82–0.88)	<0.001	0.83 (0.80–0.86)	<0.001
Cisplatin	0.86 (0.83–0.90)	<0.001	0.93 (0.89–0.97)	0.002	0.92 (0.87–0.97)	0.001
Paclitaxel	0.82 (0.78–0.85)	<0.001	0.87 (0.83–0.92)	<0.001	0.89 (0.84–0.94)	<0.001
Carboplatin	0.85 (0.79–0.90)	<0.001	0.88 (0.82–0.94)	<0.001	0.88 (0.81–0.95)	0.001
Cancer treatment						
CT	0.79 (0.76–0.82)	< 0.001	0.88 (0.83–0.93)	< 0.001	0.90 (0.84–0.96)	0.003
RT	0.93 (0.90–0.96)	< 0.001	0.96 (0.93–1.00)	0.041	0.98 (0.94–1.02)	0.23
CT regimens before EGFR-TKI						
0	1.0 (reference)		1.0 (reference)		1.0 (reference)	
1	1.04 (1.00–1.09)	0.07	1.14 (1.07–1.20)	<0.001	1.13 (1.06–1.20)	<0.001
≥2	0.96 (0.92–1.00)	0.046	1.17 (1.09–1.25)	<0.001	1.18 (1.09–1.27)	<0.001
Concomitant drug						
Statin	0.86 (0.83–0.90)	<0.001	0.85 (0.81–0.90)	<0.001	0.85 (0.80–0.89)	<0.001
NSAID	1.18 (1.10–1.27)	<0.001	1.23 (1.14–1.33)	<0.001	1.15 (1.06–1.25)	0.001
Aspirin	0.94 (0.91–0.98)	0.003	0.86 (0.82–0.89)	<0.001	0.85 (0.81–0.89)	<0.001
Anti-HTN	0.92 (0.89–0.96)	<0.001	0.84 (0.81–0.88)	<0.001	0.84 (0.81–0.88)	<0.001
Steroid	1.00 (0.96–1.04)	0.99	1.07 (1.02–1.12)	0.003	1.07 (1.02–1.12)	0.011

^a^: Model adjusted age, gender, premium level, comorbidities, anti-neoplastic agents, cancer treatments, CT regimens before EGFR-TKI and concomitant drug. Abbreviations: EGFR, epidermal growth factor receptor; TKI, tyrosine kinase inhibitor; SD, standard deviation; COPD, chronic obstructive pulmonary disease; NTD, new Taiwan dollar; CHF, congestive heart failure; DM, diabetes mellitus; CCI, Charlson comorbidity index; CT, chemotherapy; RT, radiotherapy; NSAID, non-steroidal anti-inflammatory drug; and HTN, hypertension.

**Table 3:** Comparison of HRs of progression-free survival for all covariates.

	Crude Model		Adjusted Model ^a^		Propensity Score Matched Adjusted Model	
	HR (95% CI)	*p* Value	HR (95% CI)	*p* Value	HR (95% CI)	*p* Value
Progression-free survival						
COPD	1.11 (1.08–1.15)	<0.001	1.05 (1.02–1.09)	0.004	1.05 (1.02–1.09)	0.006
Erlotinib	1.11 (1.08–1.15)	<0.001	0.36 (0.34–0.39)	<0.001	0.36 (0.33–0.39)	<0.001
Gefitinib	0.54 (0.52–0.56)	<0.001	0.28 (0.26–0.30)	<0.001	0.29 (0.26–0.31)	<0.001
Age (mean, SD), years	1.00 (1.00–1.00)	0.3	1.00 (1.00–1.00)	0.73	1.00 (1.00–1.00)	0.09
Gender						
Male vs. Female	1.50 (1.45–1.55)	<0.001	1.36 (1.31–1.41)	<0.001	1.35 (1.30–1.41)	<0.001
Premium level (NTD)						
>25,000	1.0 (reference)		1.0 (reference)		1.0 (reference)	
25,000–15,840	1.08 (1.01–1.15)	0.026	1.23 (1.15–1.32)	<0.001	1.22 (1.13–1.31)	<0.001
<15,840	1.21 (1.14–1.28)	<0.001	1.23 (1.16–1.31)	<0.001	1.21 (1.14–1.30)	<0.001
Low	1.13 (1.09–1.18)	<0.001	1.16 (1.12–1.21)	<0.001	1.16 (1.11–1.21)	<0.001
Comorbidity						
CHF	1.10 (1.02–1.18)	0.019	1.12 (1.03–1.21)	0.006	1.10 (1.00–1.22)	0.05
DM	1.05 (1.01–1.10)	0.02	1.11 (1.06–1.16)	<0.001	1.10 (1.05–1.15)	<0.001
Pneumonia	1.21 (1.16–1.26)	<0.001	1.15 (1.11–1.20)	<0.001	1.16 (1.10–1.21)	<0.001
Sepsis	1.24 (1.05–1.46)	0.011	1.10 (0.93–1.30)	0.25	1.15 (0.96–1.39)	0.13
CCI score	1.02 (1.01–1.02)	<0.001	1.02 (1.01–1.02)	<0.001	1.02 (1.01–1.02)	<0.001
Anti-neoplastic agents						
Gemcitabine	1.45 (1.40–1.49)	<0.001	1.24 (1.19–1.30)	<0.001	1.22 (1.17–1.28)	<0.001
Docetaxel	1.33 (1.29–1.38)	<0.001	1.09 (1.05–1.14)	<0.001	1.08 (1.03–1.13)	0.001
Pemetrexed	0.99 (0.96–1.02)	0.54	0.96 (0.92–1.00)	0.032	0.95 (0.91–0.99)	0.02
Vinorelbine	1.24 (1.20–1.28)	<0.001	1.11 (1.07–1.15)	<0.001	1.09 (1.05–1.14)	<0.001
Cisplatin	1.10 (1.06–1.14)	<0.001	0.90 (0.86–0.95)	<0.001	0.89 (0.84–0.94)	<0.001
Paclitaxel	1.22 (1.16–1.27)	<0.001	1.07 (1.02–1.12)	0.006	1.08 (1.02–1.14)	0.006
Carboplatin	1.08 (1.01–1.15)	0.029	0.92 (0.86–0.99)	0.021	0.92 (0.85–0.99)	0.029
Cancer treatment						
CT	1.43 (1.38–1.49)	<0.001	1.01 (0.95–1.08)	0.74	1.04 (0.97–1.11)	0.29
RT	1.13 (1.09–1.16)	<0.001	1.03 (0.99–1.07)	0.11	1.05 (1.01–1.09)	0.024
CT regimens before EGFR-TKI						
0	1.0 (reference)		1.0 (reference)		1.0 (reference)	
1	1.65 (1.58–1.72)	<0.001	1.24 (1.17–1.31)	<0.001	1.22 (1.15–1.30)	<0.001
≥2	1.93 (1.85–2.01)	<0.001	1.31 (1.22–1.40)	<0.001	1.33 (1.23–1.43)	<0.001
Concomitant drug						
Statin	0.82 (0.79–0.86)	<0.001	0.86 (0.82–0.91)	<0.001	0.86 (0.82–0.91)	<0.001
NSAID	1.41 (1.31–1.52)	<0.001	1.32 (1.23–1.43)	<0.001	1.22 (1.12–1.33)	<0.001
Aspirin	0.91 (0.87–0.94)	<0.001	0.89 (0.85–0.93)	<0.001	0.88 (0.84–0.93)	<0.001
Anti-HTN	0.95 (0.92–0.98)	0.004	0.93 (0.89–0.96)	<0.001	0.93 (0.89–0.97)	0.002
Steroid	1.28 (1.23–1.33)	<0.001	1.06 (1.02–1.11)	0.007	1.07 (1.02–1.13)	0.006

^a^: Model adjusted age, gender, premium level, comorbidities, anti-neoplastic agents, cancer treatments, CT regimens before EGFR-TKI and concomitant drug. Abbreviations: EGFR, epidermal growth factor receptor; TKI, tyrosine kinase inhibitor; SD, standard deviation; COPD, chronic obstructive pulmonary disease; NTD, new Taiwan dollar; CHF, congestive heart failure; DM, diabetes mellitus; CCI, Charlson comorbidity index; CT, chemotherapy; RT, radiotherapy; NSAID, non-steroidal anti-inflammatory drug; and HTN, hypertension.
